# Minding the gap between cortisol levels measured with second-generation assays and current diagnostic thresholds for the diagnosis of adrenal insufficiency: a single-center experience

**DOI:** 10.1007/s42000-020-00185-y

**Published:** 2020-03-28

**Authors:** G. Grassi, V. Morelli, F. Ceriotti, E. Polledri, S. Fustinoni, S. D’Agostino, G. Mantovani, I. Chiodini, M. Arosio

**Affiliations:** 1 Endocrinology Unit, Fondazione IRCCS Ca’ Granda Ospedale Maggiore Policlinico, Milan, Italy; 2 Università degli studi di Milano, Milan, Italy; 3 Clinical Laboratory, Fondazione IRCCS Ca’ Granda Ospedale Maggiore Policlinico, Milan, Italy; 4 Toxicology Unit, Fondazione IRCCS Ca’ Granda Ospedale Maggiore Policlinico, Milan, Italy; 5 Department of Clinical Sciences and Community Health, University of Milan, Milan, Italy; 6 Unit for Bone Metabolism Diseases and Diabetes & Lab of Endocrine and Metabolic Research IRCCS Istituto Auxologico Italiano, Milan, Italy

**Keywords:** Adrenal insufficiency, Cortisol assays, ACTH test, Liquid chromatography tandem mass spectrometry

## Abstract

**Purpose:**

The current cut-offs for the diagnosis of adrenal insufficiency (AI) have been established using outdated immunoassays. We compared the cortisol concentrations measured with Roche Cortisol I (R1), the newly available Roche Cortisol II (R2), and liquid chromatography tandem mass spectrometry (LC-MS/MS), the gold standard procedure to measure steroids in patients undergoing the corticotropin (ACTH) test.

**Methods:**

We enrolled 30 patients (age 47 ± 21 years) referred to undergo the ACTH test (1 or 250 μg). Cortisol was measured at 0, 30, and 60 min after stimulation with R1, R2, and LC-MS/MS. AI was diagnosed for R1-stimulated peak cortisol concentrations < 500 nmol/L.

**Results:**

Mean cortisol concentrations measured with R2 and LC-MS/MS were comparable, while mean cortisol concentrations measured by R1 were higher than those of both R2 and LC-MS/MS (respectively, basal 411 ± 177, 287 ± 119, and 295 ± 119 nmol/L; at 30 min, 704 ± 204, 480 ± 132, and 500 ± 132 nmol/L; at 60 min, 737 ± 301, 502 ± 196, and 519 ± 201 nmol/L, *p* ≤ 0.01 for R1 vs. both R2 and LC-MS/MS at each point). Considering the 500 nmol/L cortisol peak cut-off, AI was diagnosed in 5/30 patients using R1 and in 12/30 using R2 (+ 140%). Based on the correlation between R1 and R2, the threshold of 500 nmol/L became 351 nmol/L (12.7 μg/dL) when cortisol was measured with R2, and 368 nmol/L (13.3 μg/dL) with LC-MS/MS.

**Conclusions:**

The use of more specific cortisol assays results in lower cortisol concentrations. This could lead to misdiagnosis and overtreatment when assessing AI with the ACTH test if a different cut-off for cortisol peak is not adopted.

## Introduction

Adrenal insufficiency (AI) is a potentially life-threatening medical condition in which cortisol secretion is impaired because of adrenal (primary adrenal insufficiency, PAI) or pituitary failure (secondary adrenal insufficiency, SAI). Although morning serum cortisol concentrations are helpful to determine hypothalamic-pituitary-adrenal axis function, the evidence in the literature is as yet insufficient to support the use of basal cortisol concentrations alone in order to either rule out or confirm AI [1–5]. Diagnosis is mainly based on measuring cortisol concentrations after a stimulation test: the standard dose (250 μg) corticotropin (1–24 ACTH) test (SDCT) represents the gold standard dynamic test for PAI, while the insulin tolerance test (ITT) is the gold standard if SAI is suspected [1–5]. However, ITT is cumbersome and contraindicated in many patients. SDCT and the low-dose (1 μg) corticotropin test (LDCT) have been validated against ITT, and they are the most widely used tests in clinical practice because of their higher safety and tolerability [6–8]. Interpreting cut-off peak values after stimulation is also challenging [9]. Traditionally, cortisol peak concentrations > 500 nmol/L (18.1 μg/dL) are accepted in the guidelines as evidence of appropriate cortisol secretion, even if the cut-off values for exclusion of AI vary widely in different studies, from 418 nmol/L (15.2 μg/dL) to 750 nmol/L (27.1 μg/dL) according to the sensitivity/specificity chosen, timing of the peak (30 vs. 60 min), and assay used [10–13]. It must be emphasized that the thresholds for the need for replacement therapy have been established using mainly outdated radioimmunoassays, whose main limitation was the interference caused by compounds with structural similarity to the target molecule (e.g., endogenous steroids, drugs, or natural products) due to the use of polyclonal antibodies. Liquid and gas chromatography tandem mass spectrometry (LC-MS/MS and GC-MS/MS) are currently considered the gold standard procedures for cortisol measurement because they have demonstrated low cross-reactivity with other steroids; however, they are still not widely available [10]. Recently, a highly specific monoclonal and more easily available immunoassay by Roche, cortisol II (R2), calibrated on GC-MS/MS, has been released [14–18]. Using the R2 assay, new peak cortisol cut-offs as low as 375 and 351 nmol/L (13.6 and 12.7 μg/dL, respectively) have been proposed for the diagnosis of AI by two preliminary studies [17, 18]. These values are notably lower than the cut-offs suggested by the current guidelines and, thus, need to be updated. This issue is crucial for patients because of the diagnostic and therapeutic implications.

The aim of this study was to measure cortisol concentrations after ACTH stimulation in a cohort of patients referred to our center for suspected AI and to compare the results obtained with old and new assays. We evaluated how the traditional diagnostic thresholds applied to second-generation assays influence the number of AI diagnoses.

## Material and methods

### Subjects

In our study, we enrolled 30 consecutive adult patients referred to the Endocrinology Unit at Fondazione IRCCS Ca’ Granda Ospedale Maggiore Policlinico, Milan, Italy, for suspected AI to undergo the ACTH test during a period of 3 months. Pediatric patients and subjects with liver or renal impairment, or with abnormal albumin concentrations, or taking estrogen or other drugs that could interfere with cortisol secretion or dosage were excluded. Approval from the institutional review board was not required for this study because it was considered a routine activity focused on the possibility of introducing a new technology and aligning different methods, for which no extra biological samples or sensitive data were necessary.

### Methods

In all the subjects studied, we performed the ACTH test in the fasting state between 08.00 and 09.00 a.m. The patients lay supine on a bed with an intravenous catheter inserted in a forearm vein and kept clean by slow saline infusion for 30 min (basal condition). For SDCT, 1 mL of cosyntropin (Synacthen© 0.25 mg/mL, Sigma-Tau) was injected as a bolus intravenously. A 1-μg dose was prepared just before the administration as follows: 1 mL of Synacthen© 0.25 mg/mL was injected into 249 mL of sterile 0.9% saline, yielding a 1 μg/mL solution for administration. Blood serum samples were taken in the basal condition and 30 and 60 min after stimulation. In our center, the indication for cortisol replacement therapy was set at R1 stimulated peak cortisol concentrations < 500 nmol/L measured by R1, in accordance with the literature and with our experience [1–3, 11]. Although partial AI was a possibility, by using the 500-nmol/L cut-off after stimulation, we had no reported adrenal crisis in our series.

### Assays

All hormone measurements were performed in the same laboratory.

Serum cortisol concentrations were measured by two assays of the same manufacturer measured on the same analyzer (Elecsys Cortisol immunoassay, Roche Diagnostics, Mannheim, Germany, on CobaS E 602): R1, a first-generation electrochemiluminescence polyclonal immunoassay (ECLIA) with an inter-assay CV ranging from 1.4 to 2.8%, an intra-assay CV ranging from 1.0 to 1.7% and lower detection limit of 0.5 nmol/L, and R2, the second-generation monoclonal immunoassay Elecsys Cortisol II with limit of detection 1.5 nmol/L, limit of quantitation 2.0 nmol/L, inter-assay CV ranging from 1.9 to 10.1% and intra-assay CV from 1.5 to 5.4%.

Serum cortisol concentrations were also measured by LC-MS/MS using a IVD-MS steroids in serum kit (MassChrom, Steroids in Serum/Plasma, Chromsystems, Gräfelfing, Germany). Chromatographic separation and mass spectrometric detection of samples were performed with high-performance liquid chromatography (HPLC, Shimadzu, Milano, Italy) interfaced with a Sciex 4500 MD mass spectrometer (Sciex, Milano, Italy). Samples were prepared according to the manufacturer’s instructions. In short, 500 μL of each sample, calibrators, or serum quality control (QC) was placed in a solid phase extraction sample plate previously equilibrated; 50 μL of a deuterated internal standard mix solution, as well as 450 μL extraction buffer, was added. The sample plate was then vortexed and centrifuged. The extraction media was evaporated under nitrogen to dryness, reconstituted, and directly injected into an HPLC system equipped with an analytical column (operating at 32 °C) for peak separation. Mobile phases A and B (provided with the kit) were used for the chromatographic separation. For calibration, a blank calibrator matrix and six multilevel serum calibrators, provided with the kit, were used. To assess within- and between-run precision and accuracy, three certified serum QCs provided with the kit were used; these were certified by the National Institute of Standards and Toxicology (NIST) in order to promote international standardization. The limit of quantitation provided by the kit was 4 nmol/L, while our inter-assay CV ranged from 4.0 to 4.4% and intra-assay CV from 1.1 to 3.3%, based on three replicate and three analytical sessions (*N* = 12). During routine analyses, calibration curves and QCs were run within each set of unknown samples. A typical analytical sequence consisted of a calibration curve, followed by unknown samples, with QC samples repeated every ten unknown samples, followed by a second calibration curve.

### Statistical analysis

Statistical analysis was performed by SPSS version 25.0 statistical package (SPSS Inc., Chicago, IL, USA), and MedCalc statistical software for Windows (Ostend, Belgium). Comparison of continuous variables was performed using the *t* test or Mann-Whitney test, as appropriate. The normality of distribution was checked by the Kolmogorov-Smirnov test. Variables were expressed as mean±standard deviation if normally distributed, and as median (interquartile range) if not normally distributed; the comparison was performed using one-way Student’s *t* test or the Mann-Whitney *U* test, respectively, as appropriate. R1, R2, and LC-MS/MS cortisol concentrations were compared with a nonparametric Passing and Bablok regression analysis, a Cusum test, and Spearman correlation coefficients [19]. The Bland-Altman approach was used to assess differences between the two methods by plotting the percentage difference between the two assays vs. the mean concentration [20]. A *p* value less than 0.05 was considered significant. Using the obtained equations, we calculated the R2 and LC-MS/MS concentrations of cortisol corresponding to the R1 cortisol concentration of 500 nmol/L.

## Results

Fifteen patients (12/3 F/M, age 44.6 ± 24.8 years) underwent SDCT for suspected non-classical congenital adrenal hyperplasia (NCCAH) (12/15) or suspected PAI following unilateral adrenalectomy (3/15). The remaining 15 patients (12/3 F/M, age 50.9 ± 16.7 years) underwent LDCT for suspected SAI in an incidental radiological finding of empty sella (1/15) or pituitary adenoma (10/15), or after 2 months following (4/15) pituitary surgery. At the time of testing, six patients were on glucocorticoid replacement therapy (cortisone acetate, three patients as prophylaxis following adrenal surgery and three patients because of a previous diagnosis of SAI). Cortisone acetate mean dose was 25.0 ± 12.9 mg/day and mean time on replacement therapy 66.3 ± 70.4 months; all patients were on drug wash-out for 24 h before the test.

As expected, in the majority of patients undergoing LDCT, cortisol peak occurred at 30 min, and in the majority of patients undergoing SDCT, at 60 min, regardless of the cortisol measurement method used. Overall mean cortisol concentrations measured with R1, R2, and LC-MS/MS were, respectively, 411 ± 177 (14.9 ± 6.4), 287 ± 119 (10.4 ± 4.3), and 295 ± 119 (10.7 ± 4.3) nmol/L (μg/dL) in basal conditions; 704 ± 204 (25.5 ± 7.4), 480 ± 132 (17.4 ± 4.8), and 500 ± 132 (18.1 ± 4.8) nmol/L (μg/dL) at 30 min; and 737 ± 301 (26.7 ± 10.9), 502 ± 196 (18.1 ± 7.1), and 519 ± 201 (18.8 ± 7.3) nmol/L (μg/dL) at 60 min after the ACTH test. Cortisol levels measured with different methods in the basal and stimulated condition after LDCT and SDCT are reported in Table 1. The R1 cortisol concentrations were significantly higher than those of R2 and LC-MS/MS at each time point (*p* < 0.001), while cortisol concentrations obtained with R2 and LC-MS/MS using both tests were comparable (Table 1).

**Table 1:** Biochemical evaluations at basal and stimulated conditions

	Time	Cortisol R1 (nmol/L)	Cortisol R2 (nmol/L)	Cortisol LC-MS/MS (nmol/L)	*p* value (R1 vs. R2 and LC-MS/MS)
LDCT *N* = 15	0′	306 (193)	212 (102)	229 (116)	< 0.001
	30′	599 (315)	414 (163)	436 (199)	< 0.001
	60′	458 (386)	317 (229)	378 (215)	< 0.001
SDCT *N* = 15	0′	516 (348)	348 (190)	359 (182)	< 0.001
	30′	789 (190)	538 (124)	552 (119)	< 0.001
	60	927 (163)	635 (116)	646 (168)	< 0.001

*LDCT*, low-dose (1 μg) ACTH test; *SDCT*, standard dose (250 μg) ACTH test; *R1*, Roche I; *R2*, Roche II cortisol assay; *LC-MS/MS*, liquid chromatography tandem mass spectrometry; *AI*, adrenal insufficiency. Cortisol levels are expressed as median (interquartile range); categorical variables are expressed as number (percentage). Conventional unit conversion factors, SI cortisol levels/27.6

**Fig. 1:**
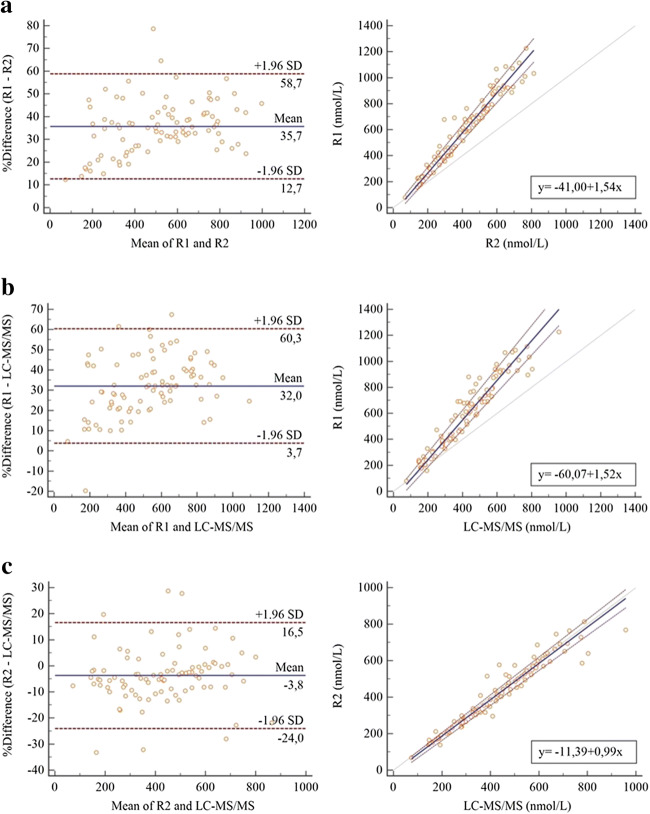
Bland-Altman plots to identify relative differences between R1 and R2 (panel **a**); R1 and LC-MS/MS (panel **b**); and R2 and LC-MS/MS (panel **c**). Mean differences are represented with solid blue lines, and 95% limits of agreement are represented with dashed lines. Passing-Bablok correlation between the serum levels of cortisol assessed by R2 vs. R1 (panel **a**); LC-MS/MS vs. R1 (panel **b**); and LC-MS/MS vs. R2 (panel **c**). The correlation lines are represented with solid blue lines, and the 95% limits of agreement are represented with dashed lines. The dotted lines indicate lines of identity

For R1 vs. R2, Passing-Bablok intercept of the linear regression was − 41.00 (95% CI: − 68.98; − 11.30) and the slope was 1.54 (95% CI: 1.46; 1.61) (Table 2). Considering R2 as the reference method, these results indicate the presence of a constant bias, as well as a proportional bias, in R1, for which values, according to the Bland-Altman plot, were approximately 36% higher (from 12.7 to 58.7%). A similar discrepancy was observed over the whole range of concentrations tested (Fig. 1a). The same response was obtained for R1 vs. LC-MS/MS, in which the Passing-Bablok intercept of the linear regression was − 60.07 (95% CI: − 98.60; − 24.53) and the slope was 1.52 (95% CI: 1.43; 1.62) (Table 2). Considering LC-MS/MS as the reference method, these results indicate the presence of both a constant and a proportional bias in R1, whose values, according to the Bland-Altman plot, were approximately 32% higher (from 3.7 to 60.3%). A similar discrepancy was observed over the whole range of concentrations tested (Fig. 1b). For R2 vs. LC-MS/MS, Passing-Bablok intercept of the linear regression was − 11.39 (95% CI: − 28.23; − 0.42) and the slope was 0.99 (95% CI: 0.96; 1.03) (Table 2). Considering LC-MS/MS as the reference method, these results indicate the absence of a proportional bias and the presence of a small constant bias for R2, whose values, according to the Bland-Altman plot, were approximately 4% lower than those of LC-MS/MS (from − 24.0 to 16.5%). A similar discrepancy was observed over the whole range of concentrations tested (Fig. 1c).

**Table 2:** Passing-Bablok correlation parameters

	Passing-Bablok regression equation	95% CI of the intercept	95% CI of the slope	Cusum test for linearity	Spearman correlation coefficient (significance)
R1 vs. R2	*y* = − 41.00 + 1.54*x*	− 68.98; − 11.30	1.46–1.61	N.S. deviation from linearity (*p* = 0.991)	0.972 (*p* < 0.001)
R1 vs. LC-MS/MS	*y* = − 60.07 + 1.52*x*	− 98.60; − 24.53	1.43–1.62	N.S. deviation from linearity (*p* = 0.635)	0.960 (*p* < 0.001)
R2 vs. LC-MS/MS	*y* = − 11.39 + 0.99*x*	− 28.23; − 0.42	0.96–1.03	N.S. deviation from linearity (*p* = 0.459)	0.973 (*p* < 0.001)

*R1*, Roche I; *R2*, Roche II cortisol assay; *LC-MS/MS*, liquid chromatography tandem mass spectrometry

Based on the correlation between R1 and R2 cortisol concentrations, we calculated that the diagnostic threshold for AI would be 351 nmol/L (12.7 μg/dL) if cortisol levels were measured with R2 (Fig. 1a). Similarly, applying the correlation between R1 and LC-MS/MS cortisol concentrations, we found that the cut-off for diagnosing AI would be 368 nmol/L (13.3 μg/dL) (Fig. 1b).

With the traditional 500 nmol/L (18 μg/dL), cut-off AI would have been diagnosed in 5/30 patients (17%) using R1, and 12/30 (40%) using R2, leading to a relevant increase in AI diagnosis in our patient cohort (+ 140%). Clinical and biochemical characteristics of the seven patients diagnosed as AI by R2 but not by R1 are shown in Table 3.

**Table 3:** Clinical and biochemical characteristics of patients diagnosed as AI by R2 but not by R1

Patient	Age	Sex	Diagnosis	Test type	Ongoing GC replacement	R1 (nmol/L)			R2 (nmol/L)			TM (nmol/L)		
						0′	30′	60′	0′	30′	60′	0′	30′	60′
P1	76	M	Unilateral adrenalectomy for subclinical hypercortisolism by adrenal adenoma	SDCT	Yes	516	585	596	403	417	442	425	477	483
P2	61	M	GH-deficiency + hypogonadism + hypothyroidism following pituitary surgery	LDCT	No	425	704	657	259	453	419	276	508	444
P3	40	F	Microprolactinoma	LDCT	No	248	599	475	201	414	359	218	436	384
P4	75	M	GH-deficiency + hypogonadism following pituitary surgery	LDCT	No	306	638	577	207	464	386	229	472	400
P5	50	F	Microprolactinoma	LDCT	No	268	541	395	212	378	293	174	223	320
P6	65	F	Non-classical congenital adrenal hyperplasia	SDCT	No	546	679	693	326	295	353	337	408	373
P7	55	F	Partial empty sella	LDCT	No	389	690	458	237	461	317	271	527	378

*AI*, adrenal insufficiency; *LDCT*, low-dose (1 μg) ACTH test; *SDCT*, standard dose (250 μg) ACTH test; *GC*, glucocorticoids

*R1*, Roche I cortisol assay; *R2*, Roche II cortisol assay; *LC-MS/MS*, liquid chromatography tandem mass spectrometry. *p < 0.001 for R1 vs. both R2 and LC-MS/MS cortisol concentrations. Conventional unit conversion factors, SI cortisol levels/27.6

## Discussion

The main findings of our study are that R2 cortisol concentrations were significantly lower than those of R1 and much more similar to those obtained by LC-MS/MS. By using the current cut-off of 500 nmol/L, we would have overestimated the presence of AI in our population, with consequent overtreatment by non-indicated hydrocortisone replacement therapy. Based on the correlation between R1 and R2 cortisol concentrations, the threshold of 500 nmol/L became 351 nmol/L (12.7 μg/dL) when cortisol levels were measured with R2 and 368 nmol/L (13.3 μg/dL) with LC-MS/MS. These apparently low levels should not be surprising if we consider that the mean R2 cortisol concentrations were about 36% lower than those of R1 at all time points. Considering LC-MS/MS as the reference method, the Bland-Altman plot and the Passing and Bablok regression analysis highlighted better performance for R2 compared with R1. This result confirms the strong correlation between R2 and LC-MS/MS, as previously reported [16, 17].

These values are fully in agreement with those recently found by Raverot and Kline [17, 18]. Raverot et al. analyzed samples from 109 patients undergoing the ACTH test (dose not specified) and ITT by ROC curves, using the 500-nmol/L cut-off for cortisol peak assessed by R1 as reference. They found a 374-nmol/L (13.6 μg/dL) threshold for R2 to have 100% sensitivity and 93.3% specificity, whereas a cut-off of 350 nmol/L (12.7 μg/dL) would enable 100% specificity, reducing sensitivity to 85% [17]. Kline et al. studied 82 patients undergoing various tests—ITT, glucagon stimulation, SDCT, or LDCT—and compared cortisol peak concentrations after stimulation tests measured by R1 and R2, as performed in our study. They obtained a new peak cortisol cut-off of 351 nmol/L (12.7 μg/dL) [18].

In our study including both SDCT and LDCT, we found exactly the same cut-off of 351 nmol/L (12.7 μg/dL) for cortisol measured by R2. We also calculated the cut-off for cortisol measured by LC-MS/MS as 368 nmol/L (13.3 μg/dL), which tends to be slightly—but not significantly—higher than R2, as shown by other authors [21].

Few data are available on cortisol concentrations measured with R2 in healthy subjects; to our knowledge, the only study to date was carried out by Ueland et al. They enrolled 121 volunteers (M=F, mean age 40 years) to undergo SDCT and evaluated stimulated cortisol cut-off for R2 and LC-MS/MS (using a different spectrometer from ours). They found that the 2.5th percentile of cortisol values at 30 and 60 min following SDCT were 440 and 548 nmol/L (15.9 and 19.8 μg/dL) by R2, and 412 and 485 nmol/L (14.9 and 17.6 μg/dL) by LC-MS/MS, respectively [15].

In our study, none of the patients undergoing SDCT was diagnosed as AI when measuring cortisol levels by R1 and using the traditional cut-off. However, based on R2 cortisol concentrations and considering the cut-offs of 440 and 548 nmol/L after 30 and 60 min, respectively, AI would have been diagnosed in two patients (P1 and P6, Table 3). P1 had recently undergone unilateral adrenalectomy for a cortisol-secreting adenoma, which is often followed by transient hypoadrenalism lasting from a few months to several years [22]. P6 received a subsequent diagnosis of NCCAH with double heterozygosity for CYP21A2 mutations in c.290-13 A/C>G and c.841 G>T (leading, respectively, to 0% and 20–50% residual enzyme activity), which is potentially associated with AI. We cannot exclude that, by using R1, we would have missed the diagnosis of AI due to the assay interference with cortisol precursors.

To the best of our knowledge, there are no studies evaluating cortisol response after LDCT by R2 and LC-MS/MS in healthy subjects. It may be difficult to diagnose AI, especially when SAI is suspected, since the additional guidance based on high basal ACTH concentrations is lacking, and no tests (including ITT) correctly classify all patients [23].

The current guidelines highlight that for a correct AI diagnosis, it is essential to understand the pre-test probability of disease and the test limitations and to apply proper clinical assessment and follow-up. Nevertheless, clinicians also need reliable biochemical data. The introduction into the clinical practice of more modern methods for cortisol measurement, such as R2 that has been calibrated on GC-MS/MS, makes the interpretation of cortisol stimulatory tests even more difficult in the absence of new cut-offs. Indeed, it is well known that LC-MS/MS and GC-MS/MS, the gold standard procedures for steroid measurement due to their lower cross-reactivity with other steroids, provide lower cortisol concentrations than older assays [14, 15, 18]. As a consequence, the cortisol cut-off to diagnose AI needs to be redefined.

The main limitations of our study are the lack of a control group for both SDCT and LDCT, the low number of included patients, and the use of two different tests for the diagnosis of AI (SDCT and LDCT), which could have introduced a bias. Notwithstanding these limitations, the present data may be useful in clinical practice, since they confirm that a much lower cortisol cut-off after ACTH stimulation needs to be adopted when cortisol is measured by R2.

In conclusion, cortisol concentrations measured by the R2 method are similar to those measured by LC-MS/MS, and their use results in lower cortisol concentrations compared with R1. This could lead to AI misdiagnosis if the current cut-off of 500 nmol/L (18 μg/dL) is used. New clinically derived thresholds are not yet available, and this has to be taken into account when interpreting stimulated cortisol peak concentrations in clinical practice.
